# Supplementary material for: Nonlinear analog processing with anisotropic nonlinear films
Source: Nanophotonics. 2025 Jun 5;14(23):4099–112. doi: 10.1515/nanoph-2024-0770 (PMC12617739; doi:10.1515/nanoph-2024-0770)
Supplement: Supplementary file 1 — Supplementary Material Details [file j_nanoph-2024-0770_suppl_001.pdf]

Supplementary Information

Table of Contents

Section S1. Linear transmission spectrum of the sample – Calculations and Measurements

Section S2. Numerical modeling of second harmonic generation from a GaAs film

Section S3. SHG efficiency versus pump polarization, pump direction and pump wavelength

Section S4. Nonlinear edge detection under different crystal orientations

Section S1. Linear transmission spectrum of the sample – Calculations and measurements

The finite thickness of the GaAs slab creates weak Fabry-Pérot resonances. As discussed in the paper and in the later sections of this SI, such resonances induce a weak spectral modulation of the SHG efficiency. To verify the magnitude of these effects, we have measured the normal-incidence transmission spectrum of the device. The measurement was performed by using a broadband supercontinuum laser (Leukos, Rock 400) filtered by a tunable narrowband filter (Photon, LLTF Contrast). The transmission spectrum was obtained by sweeping the laser wavelength and measuring the power transmitted through the metasurface with a power meter.

The measured transmission spectrum ((Fig. S1a, blue solid line) confirms the presence of strong oscillations, with a transmission contrast of $\Delta T > 40\%$ for wavelengths above 900 nm. For wavelengths below ~800 nm, the transmission suddenly drops, indicating the onset of strong absorption within the GaAs. We have numerically calculated the transmission spectrum of the GaAs slab with the transfer matrix method, by using tabulated values for the permittivity of GaAs (Palik) and assuming a slab thickness thickness of t= 480 nm. The calculation (dashed red line in Fig. S1a) shows an excellent agreement with the measurements, correctly reproducing both the magnitude and period of the oscillations at long wavelengths and the onset of absorption at lower wavelengths. As expected, the transmission spectrum depends on the slab thickness (see calculations in Fig. S1b)

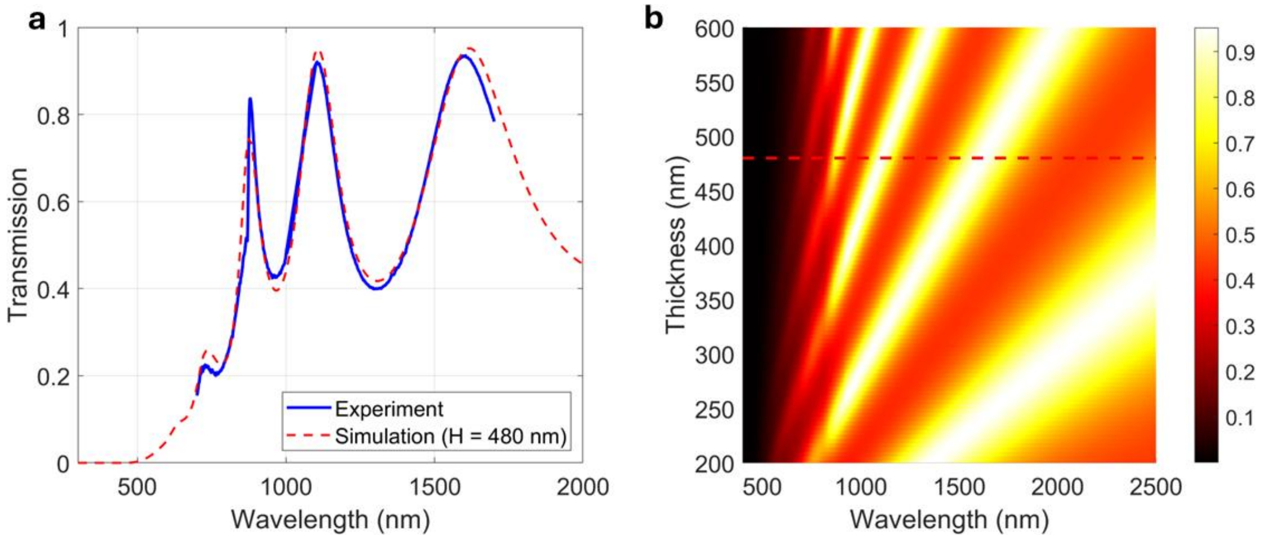

Figure S1. (a) Measured (blue solid line) and calculated (red dashed line) normal-incidence transmission spectrum of the device. In calculations, a GaAs slab thickness of t = 480 nm was assumed. **(b)** Calculated normal-incidence transmission spectrum versus slab thickness. Red dashed line denotes the spectrum shown as red dashed line in panel a.

Section S2. Numerical modeling of second-harmonic generation from a GaAs film

The simulations of second-harmonic generation (shown in the main text and in the next sections of this SI) were performed in the undepleted pump approximation using the finite-element solver of COMSOL Multiphysics in the frequency domain. First, the pump field is calculated by solving the Helmholtz equation at the fundamental frequency using a plane-wave excitation with a given elevation (polar) angle θ and azimuthal angle ϕ. Next, the pump field is used to define the polarization density source inside the GaAs slab in the Helmholtz equation for the second-harmonic frequency, $P_i^{2\omega} = \epsilon_0 \chi_{ijk}^{(2)} E_j E_k$, where i, j and k are the crystal axes of GaAs, here assumed aligned with the laboratory axes, ϵ_0 is the vacuum permittivity, $\chi_{ijk}^{(2)}$ are the entries of the nonlinear susceptibility tensor of GaAs. In crystals such as GaAs there is only one independent non-zero element of the susceptibility tensor, and the non-zero entries are those for which $i \neq j \neq k$. In other words, $\chi_{xyz}^{(2)} = \chi_{xzy}^{(2)} = \chi_{xyz}^{(2)} = \chi_{xyz}^{(2)} = \chi_{xyz}^{(2)} = \chi_{xyz}^{(2)} = \chi^{(2)}$, while all the other elements of the tensor are zero. The value of the $\chi^{(2)}$ depends on the pump wavelength. In particular, in our simulations we adopted the frequency-dependent data of $\chi^{(2)}$ reported in *S. Bergfeld, W. Daum, Phys. Rev. Lett. 90 (2003) 036801* and *M.L. Trolle, Theory of linear and nonlinear optical response: Zinc-blende semiconductors, Master's Thesis, Aalborg University, Denmark (2011)*. If the crystal axes are rotated around the [001] axis (z coordinate) by an azimuthal angle θ_c with respect to the laboratory axes, then the susceptibility is transformed by the rotation matrix $R_z(\theta_c)$, and it can be written in the laboratory frame as follows:

$$\bar{\bar{\chi}}^{(2)}(\theta_c) = \chi^{(2)} \begin{pmatrix} \{0,0,\sin 2\theta_c\} & \{0,0,\cos 2\theta_c\} & \{\sin 2\theta_c\,, \cos 2\theta_c\,, 0\} \\ \{0,0,\cos 2\theta_c\} & \{0,0,-\sin 2\theta_c\} & \{\cos 2\theta_c\,, -\sin 2\theta_c\,, 0\} \\ \{\sin 2\theta_c\,, \cos 2\theta_c\,, 0\} & \{\cos 2\theta_c\,, -\sin 2\theta_c\,, 0\} & \{0,0,0\} \end{pmatrix}$$

For the particular case of $\theta_c = \pi/4$, corresponding to the experimental conditions in the imaging experiments reported in Figs. 5-7, the non-zero tensor elements are $\chi_{xxz}^{(2)}(\pi/4) = \chi_{xzx}^{(2)}(\pi/4) = -\chi_{yyz}^{(2)}(\pi/4) = -\chi_{yzy}^{(2)}(\pi/4) = \chi_{zxx}^{(2)}(\pi/4) = -\chi_{zyy}^{(2)}(\pi/4) = \chi^{(2)}$.

Section S3. SHG efficiency versus pump polarization, pump direction and pump wavelength

In Fig. 3 of the main paper, we show the measured SHG efficiency of the sample versus pump polarization, pump direction and pump wavelength. In this section, we complement the experimental results shown in the main text with numerical simulations. Figure S2(a-b) reproduces the experimental data shown in Figs. 3(b-c) of the main paper, i.e., the measured SHG efficiency for p-polarized pump (Fig. S2a) and s-polarized pump (Fig. S2b), as a function of the pump impinging direction. The impinging direction is identified here by the polar angle θ and the azimuthal angle ϕ, following the same reference frame as in Fig. 3 of the main paper. In Figs. S2c and S2d we show the corresponding simulated data, for the same polarization and range of impinging angles. An excellent agreement between the calculated and measured data is observed. For both measurements and calculations, we normalized the SHG efficiency to the maximum value, which occur for p-polarization at $\theta = 40°$.

Due to the cubic symmetry of GaAs, the SHG efficiency is periodic with respect to the angle ϕ with a period of 90°. This is displayed in Fig. S3a, which shows the same set of calculated data as in Fig. S2c but

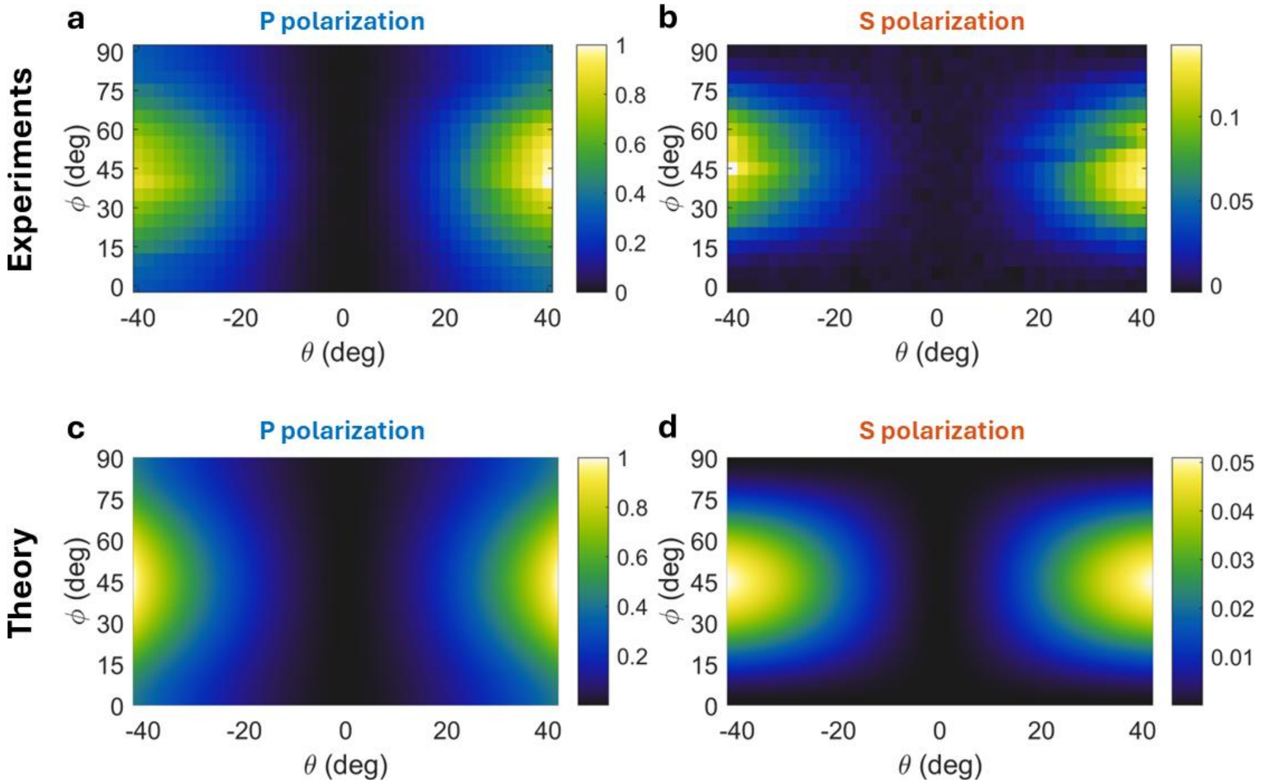

Figure S2. Measured and Simulated SHG efficiency versus pump polarization and pump impinging direction. The impinging direction is identified by the polar angle θ and the azimuthal angle ϕ, following the same reference frame as in Fig. 3 of the main paper. **(a-b)** Measured SHG efficiency versus pump direction, for p polarization (panel a) and s polarization (panel b). **(c-d)** Calculated SHG efficiency versus pump direction, for p polarization (panel c) and s polarization (panel d).

over a wider range of impinging angles. Fig. S2b shows a horizontal cross-section of Fig. S3a for $\phi = 45°$. The maximum peak for $\theta \sim 80°$ is associated with the minimum of pump reflection occurring at Brewster incidence for p-polarization.

Finally, Fig. S4a shows the calculated SHG efficiency versus pump wavelength, assuming a p-polarized pump impinging at $\theta = 40°, \phi = 45°$. As expected, the SHG efficiency peaks in the NIR region, and it is modulated by both the Fabry-Pérot resonances described above and by the intrinsic spectral dispersion of the $\chi^{(2)}$ of GaAs. For pump wavelengths below 1400 nm (corresponding to SH wavelengths below 700 nm) the SHG efficiency drops substantially due to the increased absorption of the SH inside the GaAs slab. Figure S4b shows a zoomed-in view of panel a in the range [1300 nm – 1800 nm], together with the experimentally measured efficiency (orange circles) that was reported in Fig. 3e of the main paper. Both measurements and calculations confirm the presence of a local maximum in the [1500 nm – 1600 nm] region, and a smooth decrease of the efficiency for wavelengths below or above this range.

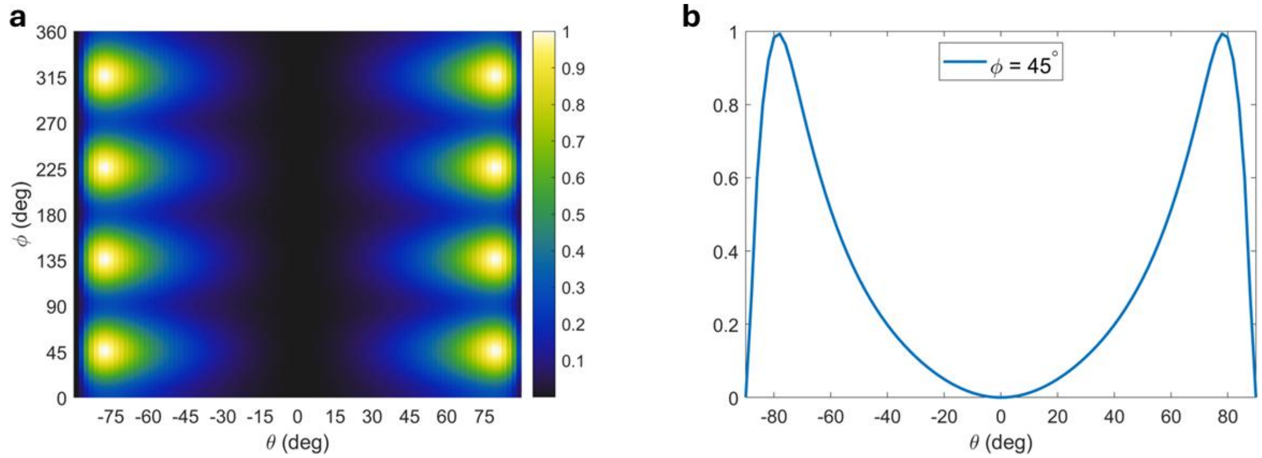

Figure S3. Calculated SHG efficiency for p-polarized pump. (a) Same data as in Fig. S2c but shown on a wider range of values of θ and ϕ. **(b)** Horizontal cross-section of panel a, for $\phi = 45°$.

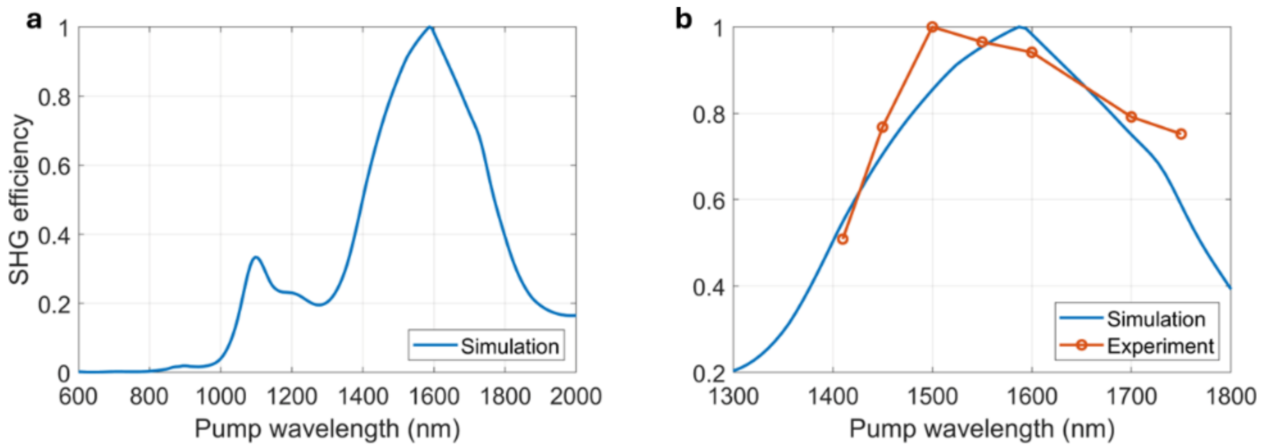

Figure S4. SHG efficiency versus wavelength. (a) Calculated SHG efficiency (in arbitrary units) for a p-polarized pump impinging at $\theta = 40°, \phi = 45°$, versus the pump wavelength. **(b)** Zoomed-in view of panel a in the [1300 nm – 1800 nm] range. The orange line corresponds to the experimentally measured efficiency reported in Fig. 3e of the main paper. Both measured and calculated efficiencies have been normalized such that their maximum is equal to 1.

Section S4. Nonlinear edge detection under different crystal orientations

Here we show how the edge detection is affected by the orientation of the GaAs crystal with respect to the basis of liner polarization of the input image. In particular, we consider edge detection under two different scenarios: (i) the crystal axes are aligned to the laboratory axes; (ii) the *xy* crystal axes are rotated by 45° with respect to the *xy* laboratory axes. For both scenarios, the edge detection is evaluated under linear and circular polarization pumps, defined with respect to the laboratory frame. As a benchmark input object, an input aperture with octagonal shape is chosen, as illustrated in Fig. S5. To better highlight the edges detected

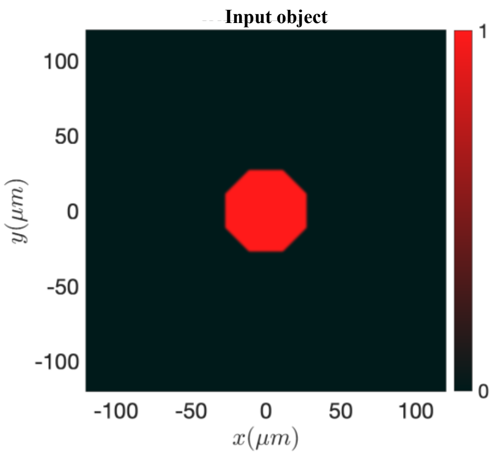

Figure S5. Input object with octagonal shape for nonlinear edge detection. An aperture with octagonal shape is used to test nonlinear edge detection under different crystal axes orientations and different pump polarization states.

at the second harmonic from the GaAs film, the octagon has smooth edges, obtained with a gaussian low-pass filter.

In Fig. S6 we show the SHG image emerging from the GaAs film in the first scenario (crystal and laboratory frames aligned). While for circularly polarized pumps all the edges of the octagon are enhanced with equal intensity, the edge detection for linearly polarized pumps shows a pronounced sensitivity to the polarization orientation, in accordance with the experimental data shown in Fig. 6 of the main text. In particular, the edges detected with largest intensity, i.e., with maximum second-harmonic generation efficiency, are those perpendicular to the pump polarization orientation. Edges parallel to the pump polarization are weakly detected. In the particular case in which the polarization axis of the pump is aligned to one of the crystal axes, the second-harmonic intensity of the edges parallel to the pump polarization vanish. The same behavior, with edge detection sensitivity rotated by 45°, can be observed in the second scenario (illustrated in Fig. S7), in which the crystal axes rotated by 45° with respect to the laboratory axes.

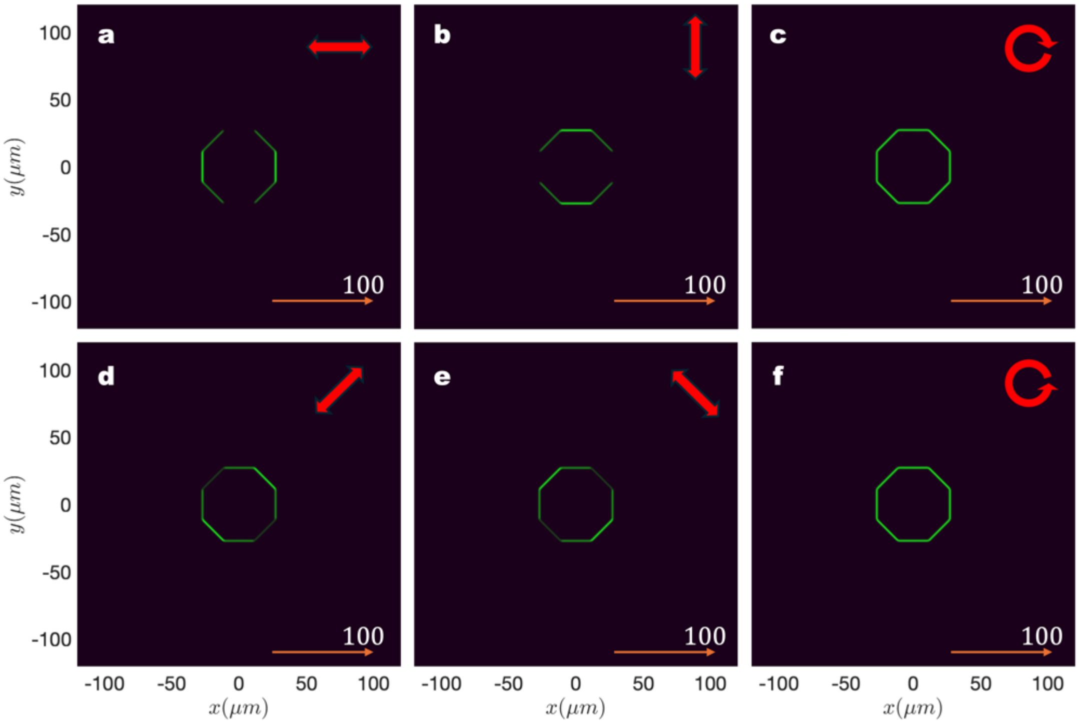

Figure S6. Nonlinear edge detection of an octagonal shape when the crystal axes are aligned to the laboratory axes. (a) Pump horizontally polarized ($\hat{x}$) and parallel to the [100] axis of the crystal. Edges along this direction are not detected. **(b)** Pump vertically polarized ($\hat{y}$) and perpendicular to the [100] axis of the crystal. Edges along this direction are not detected. **(c)** Pump with right circular polarization $[(\hat{x} + i\hat{y})/\sqrt{2}\,)]$. **(d)** Pump linearly polarized at 45° with respect to the [100] crystal axis $[(\hat{x} + \hat{y})/\sqrt{2}\,)]$. Edges along this direction are weakly detected. **(e)** Pump linearly polarized at 45° with respect to the [100] crystal axis $[(\hat{x} + \hat{y})/\sqrt{2}\,)]$. Edges along this direction are weakly detected. **(f)** Pump linearly polarized at -45° with respect to the [100] crystal axis $[(\hat{x} - \hat{y})/\sqrt{2}\,)]$. Edges along this direction are weakly detected **(f)** Pump with left circular polarization $[(\hat{x} - i\hat{y})/\sqrt{2}\,)]$.

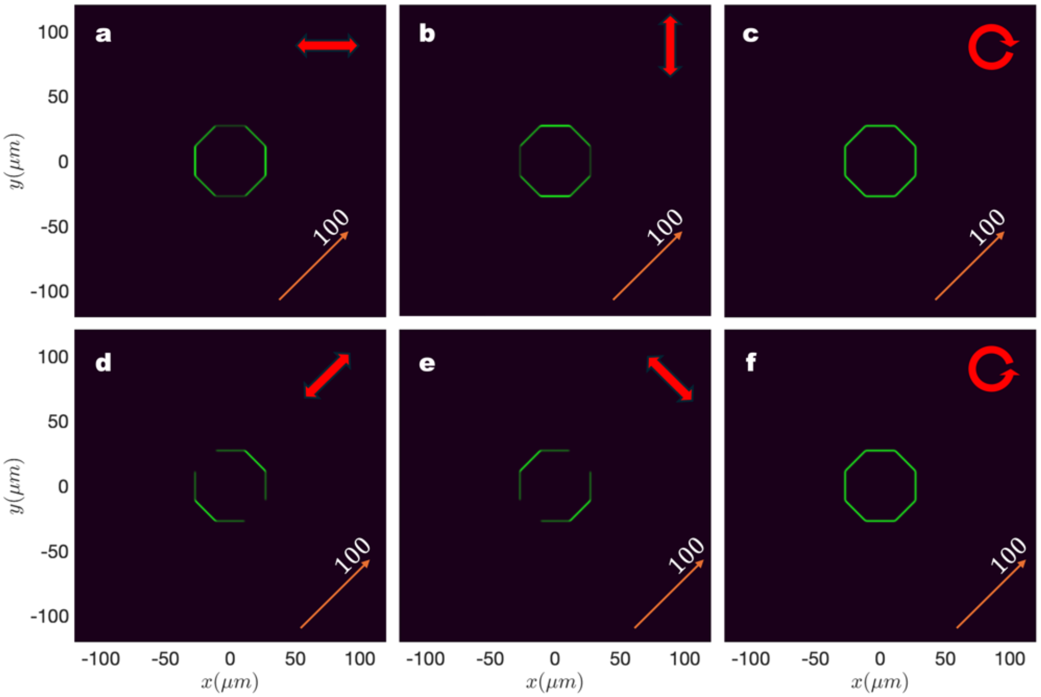

Figure S7. Nonlinear edge detection of an octagonal shape when the crystal axes are rotated by 45° with respect to the laboratory axes. (a) Pump horizontally polarized ($\hat{x}$), at -45° with respect to the [100] crystal axis. Edges along this direction are weakly detected. **(b)** Pump vertically polarized ($\hat{y}$) at +45° with respect to the [100] crystal axis. Edges along this direction are weakly detected. **(c)** Pump with right circular polarization $[(\hat{x} + i\hat{y})/\sqrt{2}\,)]$. **(d)** Pump linearly polarized at 45°, parallel to the [100] crystal axis $[(\hat{x} + \hat{y})/\sqrt{2}\,)]$. Edges along this direction are not detected. **(e)** Pump linearly polarized at -45°, perpendicular to the [100] crystal axis $[(\hat{x} - \hat{y})/\sqrt{2}\,)]$. Edges along this direction are not detected. **(f)** Pump with left circular polarization $[(\hat{x} - i\hat{y})/\sqrt{2}\,)]$.
